# A New Image Registration Algorithm Based on Evidential Reasoning

**DOI:** 10.3390/s19051091

**Published:** 2019-03-04

**Authors:** Zhe Zhang, Deqiang Han, Jean Dezert, Yi Yang

**Affiliations:** 1 MOE KLINNS Lab, Institute of Integrated Automation, School of Electronic and Information Engineering, Xi’an Jiaotong University, Xi’an 710049, China; zhangzsmg@gmail.com; 2 ONERA, The French Aerospace Lab, Chemin de la Hunière, F-91761 Palaiseau, France; jean.dezert@onera.fr; 3 SKLSVMS, School of Aerospace, Xi’an Jiaotong University, Xi’an 710049, China; jiafeiyy@mail.xjtu.edu.cn

**Keywords:** image registration, evidential reasoning, belief functions, uncertainty

## Abstract

Image registration is a crucial and fundamental problem in image processing and computer vision, which aims to align two or more images of the same scene acquired from different views or at different times. In image registration, since different keypoints (e.g., corners) or similarity measures might lead to different registration results, the selection of keypoint detection algorithms or similarity measures would bring uncertainty. These different keypoint detectors or similarity measures have their own pros and cons and can be jointly used to expect a better registration result. In this paper, the uncertainty caused by the selection of keypoint detector or similarity measure is addressed using the theory of belief functions, and image information at different levels are jointly used to achieve a more accurate image registration. Experimental results and related analyses show that our proposed algorithm can achieve more precise image registration results compared to several prevailing algorithms.

## 1. Introduction

Image registration is a fundamental problem encountered in image processing, e.g., image fusion [1] and image change detection [2]. It refers to the alignment of two or more images of the same scene taken at different time, from different sensors, or from different viewpoints. Image registration plays an increasingly important role in applications of surveillance [3], remote-sensing [4] and medical imaging [5].

For a collection of images to be registered, one is chosen as the reference image and the others are selected as sensed images. Image registration align each sensed image to the reference image by finding the correspondence between all pixels in the image pair and estimating the spatial transformation from the sensed image to the reference image. In this paper, we just consider the image registration between two images, i.e., there is only one sensed image together with a given reference image.

Current image registration techniques that based on image domain can be generally divided into two categories [6]: the sparse methods and dense methods. There are also some methods based on transform domain, like Fourier-Mellin transformation method [7]. The transform domain based methods are often used for image registration with similarity transformation model. In this paper, we focus on the image domain based methods.

The sparse methods [8] extracts and matches salient features from the reference image and sensed image and then estimates the spatial transformation between the two images based on these matched features. Line features (e.g., edges) and point features (corners, line intersections and gravities of regions) all can be used for image registration. Corner features are the mostly used features and can be manually selected or automatically detected by Harris [9], FAST (Features from Accelerated Segment Test) [10], SIFT (Scale-Invariant Feature Transform) [11], SURF (Speeded-Up Robust Features) [12], DAISY [13], ORB (Oriented FAST and Rotated BRIEF) [14], KAZE [15], etc.

In contrast to the sparse methods, the dense methods [16] do not detect features from the image pair but search the optimal spatial transformation directly that can best match all the pixels in the image pair. Similarity (resp. dissimilarity) measures are defined to quantify the independency (resp. dependency) between the pair of images. Various similarity and dissimilarity measures have been proposed [17] such as RMSE (Root-Mean-Squared Error), PSNR (Peak Signal to Noise Ratio), Spearman’s Rho [18], NCC (Normalized Cross-correlation Coefficient) and MI (Mutual Information). It should be noted that dense methods based on RMSE or PSNR cannot handle the cases with illumination variation since these two similarity/dissmilarity measures are very sensitive to illumination changes.

Both the sparse methods and dense methods involve uncertainty problems. For the sparse methods, keypoints obtained from different keypoint detectors describe different corner features of the image. Therefore, image registrations based on different keypoint detectors would obtain different spatial transformations. For the dense methods, different similarity (dissimilarity) measures quantify the difference between the pair of images from different aspects so that image registrations based on different similarity (dissimilarity) measures would obtain different spatial transformations. These different spatial transformations obtained have their own pros and cons, and the selection of the spatial transformation (the selection of the feature detector or similarity measure indeed) would bring uncertainty.

To deal with the uncertainty caused by the particular selection of feature detector or similarity (dissimilarity) measure, one feasible way is to combine these registration transformations obtained from different feature detection methods or similarity measures to obtain a better registration result. The belief functions introduced in Demspter–Shafer Theory (DST) [19] of evidence offer a powerful theoretical tool for uncertainty modeling and reasoning; therefore, we propose a fusion based image registration method using belief functions. In this paper, the spatial transformations obtained from different feature detection algorithms or similarity measures compose the frame of discernment (FOD) and their uncertainties are modeled using belief functions. In uncertainty modeling, image information at different levels, i.e., image’s intensities, edges and phase angles, are jointly used to evaluate the beliefs about image transformations. Then, these uncertainties are further handled through the evidence combination of the above multiple information. The final registration result is obtained according to the combined evidence.

This paper is an extension of our previous work in [20] where the basic idea is briefly presented. The main added values with respect to [20] are as follows. First, the transformation model between the reference image and sensed image is more comprehensive. We use similarity transformation model in [20] but use projective transformation model in this paper, which is more general since all similarity transformations are examples of projective transformations. Second, the keypoints used in the sparse approach in [20] are manually selected. To reduce the subjective influence to the registration result, in this paper, the keypoints are generated from detection algorithms. Accordingly, feature matching and mismatching removal are added after the keypoint detection. Third, when modeling uncertainties, one more information source, i.e., image’s phase angle information, is considered in this work. Fourth, more experiments and analyses are provided for performance evaluation and analysis.

The rest of this paper is organized as follows. The basics of image registration are introduced in Section 2. The basics of evidence theory are introduced in Section 4. The proposed image registration method is introduced in Section 4.1 with emphasis of uncertainty modeling and handling. Evaluation method is introduced in Section 5. Experiment results of the proposed method and other registration methods are presented and compared in Section 6.1. Concluding remarks are given in Section 7.

## 2. Basics of Image Registration

For two (or more) images of the same scene taken at different time, from different sensors, or from different viewpoints, one is chosen as the reference image (*R*) and the other one is chosen as the sensed image (*S*). In this paper, we focus on the projective transformation model between the reference image and sensed image, which is a commonly used model in image registration [16].

Denote pixel coordinates in the reference image *R* as $\left(v,w\right)$ and their mapping counterparts in the sensed image *S* as $\left(g,h\right)$. The projective transformation from *R* to *S* can be expressed based on the homogeneous coordinates (Homogeneous coordinates can easily express the translation transformation as matrix multiplications while Cartesian coordinates cannot) as

(1) $$\left[g\;h\;1\right]=\left[v\;w\;1\right]T=\left[v\;w\;1\right]\left[\begin{array}{ccc} t_{11} & t_{12} & t_{13}\\ t_{21} & t_{22} & t_{23}\\ t_{31} & t_{32} & t_{33} \end{array}\right]$$

The similarity transformation and affine transformation are important specializations of the projective transformation, as illustrated in Table 1.

The purpose of image registration is to estimate the transformation *T* to align the sensed image *S* with the reference image *R* by

(2) $$\left[v^{\prime }\;w^{\prime }\;1\right]=\left[g\;h\;1\right]T^{-1},$$

where $\left(v^{\prime },w^{\prime }\right)$ and (g,h) denote pixel coordinates in registered sensed image $S^{\prime }$ and sensed image *S*, respectively. Current image registration techniques can be divided into two categories [6] in general, including the sparse method and dense method. Basics of these two methods are introduced below.

### 2.1. Sparse Image Registration and Its Uncertainty

**Figure 1:**
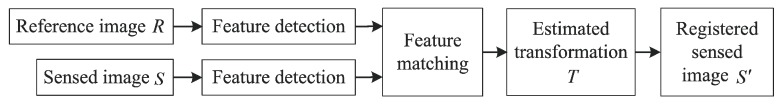
Flow chart of sparse approach.

The feature detection and feature matching are two critical steps in the sparse methods. The flow chart of the sparse approach is illustrated in Figure 1, where each functional block is detailed in the sequel.

#### 2.1.1. Feature Detection

Corner features are the mostly used features in image registration due to their invariance to imaging geometry [6]. Some early keypoint detectors, like Harris and FAST, are very sensitive to image scale changes so that have poor performance when the sensed images have different scales with the reference image. The most well-known SIFT detector shows good robustness to illumination, orientation and scale changes. Most scale invariant detectors, like SIFT, SURF, ORB and BRISK, detect and describe features at different scale levels by building or approximating the Gaussian scale space of the image. In a different way, KAZE detects features in a nonlinear scale space built using efficient additive operator splitting techniques and variable conductance diffusion.

#### 2.1.2. Feature Matching

To align the sensed image and the reference image, the detected keypoints in the two images are matched first by comparing their local feature characterized by descriptors. Generally, if the two keypoints’ descriptors are similar, the two keypoints are likely to be a matched pair. Given a keypoint *t* in the reference image, there might be a set of candidates in the sensed image having similar descriptor with *t*. Among these candidates, *t*’s real counterpart should have the closest distance with *t*, and at the same time its distance should be much closer than other candidates’ distances.

The accuracy of the keypoints’ matching affects the accuracy of the transformation’s estimation. The mismatched keypoint pairs should be further removed before estimating the transformation. RANSAC (RANdom SAmple Consensus) [21] and MSAC (M-estimator SAmple and Consensus) [22] are often used to deal with this problem. A recent RANRESAC (RANdom RESAmple Consensus) [23] algorithm has been proposed to remove mismatched keypoint pairs for noisy image registration. Besides the accuracy of the keypoints’ matching, the distribution of matched pairs over the image space is another key factor to obtain a high-quality estimation of transformation.

#### 2.1.3. Transformation Estimation

With all the matched keypoint pairs, the transformation matrix *T* can be estimated using Equation (1). Since *T* has eight degrees of freedom, four point correspondences (with no three collinear) are needed to obtain the unique solution of *T* according to Cramer’s rule.

Normally, the amount of the matched keypoint pairs is more than four and *T* can be estimated using the least squares (LS) fitting technique [6] by searching the minimum sum of the Euclidean distances between all the matched keypoints:

(3) $$\hat{T}=\arg\min\sum_{i}d\left(cor_{i}^{R},cor_{i}^{S^{\prime }}\right),$$

where $cor_{i}^{R}=\left(v_{i},w_{i}\right)$ represents the coordinate of the *i*th matched keypoint in the reference image and $cor_{i}^{S^{\prime }}=\left(v_{i}^{\prime },w_{i}^{\prime }\right)$ represents the coordinate of the *i*th matched keypoint in the registered sensed image transformed from the sensed image using Equation (2).

#### 2.1.4. Uncertainty Encountered in Sparse Approach

**Figure 2:**
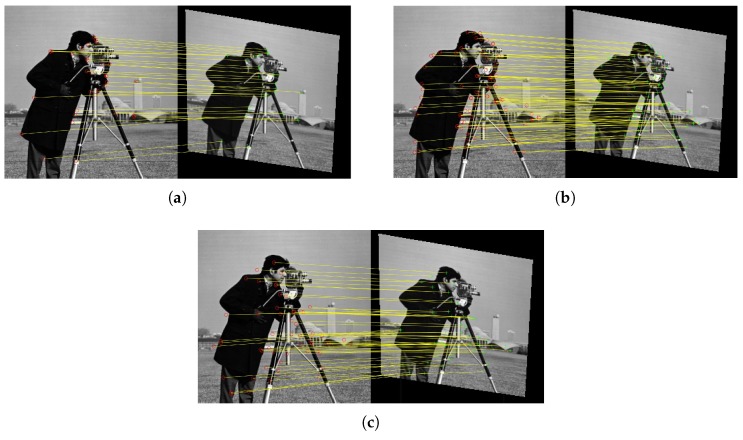
Different keypoint pairs detected by different keypoint detectors. (**a**) BRISK. (**b**) KAZE. (**c**) SURF.

Since different keypoint detection algorithms detect different kinds of corner features, the detected keypoints are usually different, as shown in Figure 2.

Image registrations based on different matched keypoint pairs would in general yield different spatial transformations to align two images. Different transformations obtained have their own pros and cons. Therefore, the selection of keypoint detection algorithms would bring uncertainty problem to the registration results.

### 2.2. Dense Image Registration and Its Uncertainty

**Figure 3:**
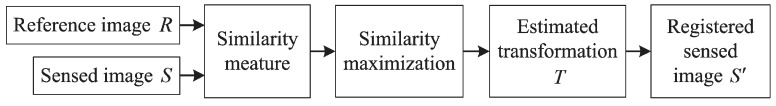
Flow chart of dense approach.

The dense image registration estimates the optimal transformation *T* by searching the largest similarity (or the smallest dissimilarity) between the reference image *R* and the registered sensed image $S^{\prime }=T\left(S\right)$:

(4) $$\hat{T}=\arg\max Sim\left(R,T\left(S\right)\right)$$

where Sim is a chosen similarity measure. The flow chart of the dense approach is illustrated in Figure 3, where each functional block is detailed in the sequel.

#### 2.2.1. Similarity Measure

Various similarity (or dissimilarity) measures have been proposed. Here we briefly introduce the commonly used MI, NCC and PSNR measures.

(1)    MI

MI measure between images *A* and *B* is

(5) $$\mathrm{MI}\left(A,B\right)=\sum_{a=0}^{255}\sum_{b=0}^{255}p_{AB}\left(a,b\right)\log\frac{P_{AB}\left(a,b\right)}{P_{A}\left(a\right)P_{B}\left(b\right)},$$

where $p_{AB}$ is the joint probability distribution function (PDF) of images *A* and *B*, and $p_{A}$ and $p_{B}$ are the marginal PDFs of *A* and *B*, respectively. MI(A,B) is larger when *A* and *B* are more similar.

(2)    NCC

For given images *A* and *B* with size of M×N, NCC measure between them is

(6) $$\mathrm{NCC}\left(A,B\right)=\sum_{x=1}^{M}\sum_{y=1}^{N}\frac{\left(A\left(x,y\right)-\mu_{A}\right)\left(B\left(x,y\right)-\mu_{B}\right)}{\sigma_{A}\sigma_{B}},$$

where A(x,y) and B(x,y) are the pixels’ intensities in images *A* and *B* at (x,y), respectively; $\mu_{A}$ and $\mu_{B}$ are the mean intensities of *A* and *B*, respectively; $\sigma_{A}$ and $\sigma_{B}$ are the standard deviation intensities of *A* and *B*, respectively. NCC(A,B) is larger when *A* and *B* are more similar.

(3)    PSNR

PSNR measure between images *A* and *B* is

(7) $$\mathrm{PSNR}\left(A,B\right)=10\times \log_{10}\left(\frac{255^{2}}{\mathrm{MSE}(A,B)}\right),$$

where MSE$\left(A,B\right)=\frac{1}{M\times N}\sum_{x=1}^{M}\sum_{y=1}^{N}{\left[A(x,y)-B(x,y)\right]}^{2}$. PSNR(A,B) is larger when *A* and *B* are more similar. Since PSNR measure is very sensitive to illumination changes, it cannot be used for image registration when there are illumination variations between image pairs.

#### 2.2.2. Transformation Estimation

The estimation for transformation *T*, i.e., Equation (4), is always a non-convex problem and is not so easy to obtain the global maximum [24]. Therefore, advanced optimization methods [25], or intelligent optimization approaches (like genetic, or particle swarm algorithms, etc.) are often used to estimate the optimal transformation *T*.

#### 2.2.3. Uncertainty Encountered in Dense Approach

Since different similarity (dissimilarity) measures compare two images from different aspects, their calculated similarities (dissimilarities) between the reference image and registered sensed image are different. Image registration based on different measures would obtain different spatial transformations to align two images and they have their own pros and cons. Therefore, the selection of similarity (dissimilarity) measure would bring uncertainty problem to the registration results.

To deal with the uncertainty caused by the selection of feature detection algorithms or similarity measures, one feasible way is to combine the registration transformations ($T_{1}$, $T_{2}$, …, $T_{Q}$) obtained from different feature detection algorithms (or different similarity measures) to expect a better registration result. We propose an evidential reasoning [19] based image registration algorithm to generate a combined transformation from $T_{1}$, $T_{2}$, …, $T_{Q}$ thanks to the ability of belief functions for uncertainty modeling and reasoning. Basics of the theory of belief functions are recalled first below.

## 3. Basics of Evidence Theory

Dempster–Shafer evidence theory (DST) [19] is a theoretical framework for uncertainty modeling and reasoning. In DST, elements in the frame of discernment (FOD) $\Theta =\{\theta_{1},\theta_{2},\ldots ,\theta_{Q}\}$ are mutually exclusive and exhaustive. The power set of Θ, i.e., $2^{\Theta }$, is the set of all subsets of Θ. For example, if $\Theta =\{\theta_{1},\theta_{2},\theta_{3}\}$, then $2^{\Theta }=\left\{\left\{\emptyset \right\},\left\{\theta_{1}\right\},\left\{\theta_{2}\right\},\left\{\theta_{3}\right\},\left\{\theta_{1},\theta_{2}\right\},\left\{\theta_{1},\theta_{3}\right\},\left\{\theta_{2},\theta_{3}\right\},\left\{\theta_{1},\theta_{2},\theta_{3}\right\}\right\}$. The basic belief assignment (BBA, also called mass function) is defined by a function *m*: $2^{\Theta }\mapsto \left[0,1\right]$, satisfying

(8) $$\sum_{A\subseteq \Theta }m(A)=1\;,\;m\left(\emptyset \right)=0,$$

where m(A) depicts the evidence support to the proposition *A*. *A* is called a focal element when m(A)>0. If there is only one element in *A*, like $\left\{\theta_{1}\right\}$ and $\left\{\theta_{2}\right\}$, *A* is called the singleton element; if there are more than one element in *A*, e.g., $\left\{\theta_{1},\theta_{2}\right\}$ and $\left\{\theta_{1},\theta_{2},\theta_{3}\right\}$, *A* is called the compound element. The belief assigned to a compound element represents the degree of ambiguity for the multiple elements.

The plausibility function (Pl) and belief function (Bel) are defined as follows:

(9) $$Pl\left(A\right)=\sum_{A\cap B\neq \emptyset }m(B),$$

(10) $$Bel\left(A\right)=\sum_{B\subseteq A}m(B).$$

Dempster’s combination rule [19] for combining two distinct pieces of evidence is defined as

(11) $$\left(m_{1}⊕m_{2}\right)\left(A\right)=\left\{\begin{array}{c} 0,\;A=\emptyset \\ \frac{1}{1-K}\sum_{B\cap C=A}\;m_{1}\left(B\right)m_{2}\left(C\right),\;A\neq \emptyset \end{array}\right.$$

Here, $K=\sum_{B\cap C=\emptyset }\;m_{1}\left(B\right)m_{2}\left(C\right)$ denotes the total conflict or contradictory mass assignments.

An alternative fusion rule PCR6 [26] for the combination of two sources is defined as

(12) $$m_{12}^{PCR6}\left(A\right)=m_{12}^{Conj}\left(A\right)+\sum_{\begin{array}{c} A\cap Y=\emptyset \end{array}}\left[\frac{m_{1}{\left(A\right)}^{2}m_{2}\left(Y\right)}{m_{1}\left(A\right)+m_{2}\left(Y\right)}+\frac{m_{2}{\left(A\right)}^{2}m_{1}\left(Y\right)}{m_{2}\left(A\right)+m_{1}\left(Y\right)}\right]$$

where $m_{12}^{Conj}\left(A\right)$ is the conjunctive rule defined as

(13) $$m_{12}^{Conj}\left(A\right)=\sum_{B\cap C=A}m_{1}\left(B\right)m_{2}\left(C\right)$$

General PCR6 formula for the combination of more than two sources is given in [26].

For a probabilistic decision-making, Smets defined the pignistic probability transformation [27] to obtain the probability measure BetP from a BBA

(14) $$BetP\left(\theta_{i}\right)\overset{\Delta }{=}\sum_{\theta_{i}\in A}\frac{m(A)}{\left|A\right|}\;\forall \theta_{i}\in \Theta ,$$

where $\left|A\right|$ is the cardinality of *A*. The decision can be made by choosing the element in FOD whose BetP value is the highest one and higher than a preset threshold. Other types of probability transformation methods can be found in [26,28].

## 4. Image Registration Based on Evidential Reasoning

To deal with the uncertainty caused by the choice of keypoint detectors in the sparse approach or the choice of similarity measure in the dense approach, we propose an image registration method based on evidential reasoning. Suppose that the spatial transformation between the reference image and sensed image is projective. Our purpose is to estimate the transformation matrix to align two images. Unlike the prevailing methods estimating the transformation matrix from single method of keypoint detection or similarity (dissimilarity) measure, we estimate the transformation matrix by jointly utilizing different keypoint detection methods or similarity measures.

**Figure 4:**
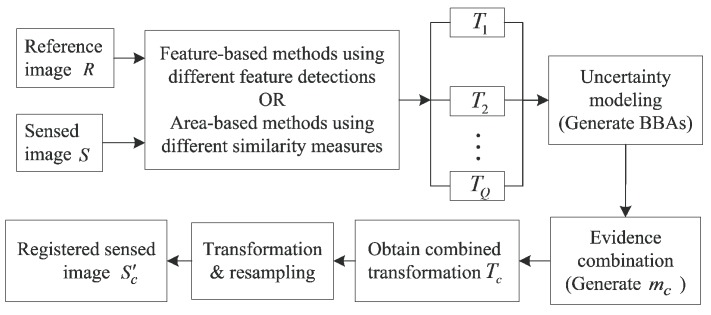
Flow chart of the proposed image registration.

To use belief functions for image registration, one should define the frame of discernment (FOD) first. The FOD $\Theta =\{T_{1},T_{2},\ldots ,T_{Q}\}$, where *Q* is the amount of transformations obtained from different single feature detection algorithms or different single similarity measures. We first model the beliefs for every proposition A⊆Θ using BBAs. *A* can be single transformation in FOD or a set of transformations in FOD. One BBA depicts the support to each proposition *A* from one evidence source. The BBA allocations from different evidence sources describes the uncertainty of the transformations in FOD. Next, the BBAs are combined to generate the combined BBA $m_{c}$ depicting the fused support to each proposition *A*. Then, the combined transformation $T_{c}$ is generated from the combined BBA $m_{c}$. Finally, the registered sensed image $S_{c}^{\prime }$ is transformed from the sensed image using Equation (2). During this process, the resampling [29] is needed to determine the intensity of each pixel in $S_{c}^{\prime }$. Figure 4 illustrates the flow chart of this new proposed method. It should be noted that the classical interpretation of BFT assumes that the final estimation should be in the FOD. In this work, we relax this assumption and the final transformation is a combination result of those in the FOD.

### 4.1. Uncertainty Modeling

If the similarity between the reference image *R* and registered sensed image ${S_{i}}^{\prime }$ is large, the corresponding transformation $T_{i}$ is quite accurate and should be allocated large support (${S_{i}}^{\prime }$ is transformed from sensed image *S* by $T_{i}^{-1}$). Here we use NCC (Other similarity or dissimilarity measures, e.g., MI, are also appropriate to quantify the similarity here) to measure the similarity between *R* and ${S_{i}}^{\prime }$:

(15) $${\mathrm{NCC}}_{i}=\sum_{x=1}^{M}\sum_{y=1}^{N}\frac{\left(R\left(x,y\right)-\mu_{R}\right)\left(S_{i}^{\prime }\left(x,y\right)-\mu_{S_{i}^{\prime }}\right)}{\sigma_{R}\sigma_{S_{i}^{\prime }}}$$

where $\mu_{R}$ and $\mu_{S_{i}^{\prime }}$ are the mean intensities of *R* and $S_{i}^{\prime }$, respectively; $\sigma_{R}$ and $\sigma_{S_{i}^{\prime }}$ are the standard deviation intensities of *R* and $S_{i}^{\prime }$, respectively.

**Figure 5:**
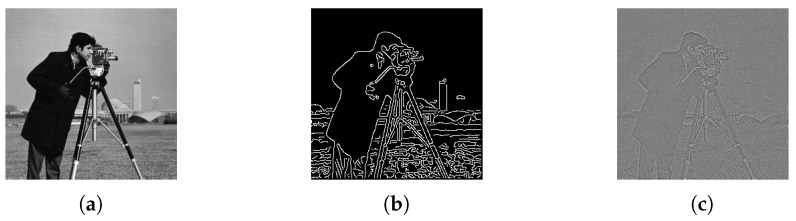
Image information at different levels. (**a**) Gray image. (**b**) Edge feature image. (**c**) Reconstructed image using phase angle.

Since multi-source information can help to reduce the uncertainty through evidence combination, we use different levels of image information to quantify the similarity between *R* and $S_{i}^{\prime }$. The similarity can be calculated from the gray images, edge feature images or reconstructed images using phase angle as shown in Figure 5. Their corresponding $NCC_{i}$ are denoted as ${\mathrm{NCC}}_{i}\left(G\right)$, ${\mathrm{NCC}}_{i}\left(E\right)$ and ${\mathrm{NCC}}_{i}\left(P\right)$, respectively. The edge detection method used in Figure 5b is the Canny detector [30]. More details of the image reconstruction from phase angle information can be found in [29].

**Figure 6:**
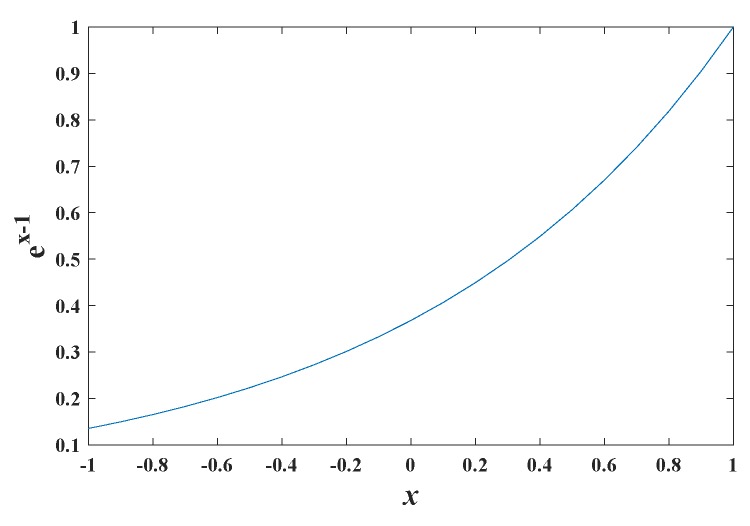
The curve of function $e^{x-1}$.

The value range of ${\mathrm{NCC}}_{i}\left(\cdot \right)$ is [−1,1]. According to our experiments, most values of ${\mathrm{NCC}}_{i}\left(\cdot \right)$ are larger than 0. Before allocating BBAs, we first enlarge the differences of ${\mathrm{NCC}}_{i}\left(\cdot \right)$ within [0,1] using function $y=e^{x-1}$, as illustrated in Figure 6.

Each level of image information (gray images (*G*), edge feature images (*E*) and reconstructed images using phase angle (*P*)) can be viewed as one evidence source and their corresponding $e^{NCC_{i}\left(\cdot \right)-1}$ can be used to assign beliefs for transformation $T_{i}$:

(16) $$\left\{\begin{array}{c} m_{G}\left(T_{i}\right)=\frac{e^{NCC_{i}\left(G\right)-1}}{\sum_{j=1}^{Q}e^{NCC_{j}\left(G\right)-1}}\\ m_{E}\left(T_{i}\right)=\frac{e^{NCC_{i}\left(E\right)-1}}{\sum_{j=1}^{Q}e^{NCC_{j}\left(E\right)-1}}\\ m_{P}\left(T_{i}\right)=\frac{e^{NCC_{i}\left(P\right)-1}}{\sum_{j=1}^{Q}e^{NCC_{j}\left(P\right)-1}} \end{array}\right.$$

### 4.2. Fusion-Based Registration

After obtaining BBAs $m_{G}$, $m_{E}$ and $m_{P}$, we generate the combined BBA $m_{c}$ using a combination rule denoted symbolically with ⊕:

(17) $$m_{c}\left(\cdot \right)=\left[m_{G}⊕m_{E}⊕m_{P}\right]\left(\cdot \right)$$

$m_{c}\left(T_{i}\right)$ describes the combined evidence support to $T_{i}$ (a 3×3 matrix with 6 unknown parameters). The combined transformation $T_{c}$ is computed by

(18) $$T_{c}^{-1}=\sum_{i=1}^{Q}m_{c}\left(T_{i}\right)T_{i}^{-1}.$$

Finally, the registered sensed image ${S_{c}}^{\prime }$ can be obtained using Equation (2) following the resampling.

## 5. Evaluation of Image Registration

**Figure 7:**
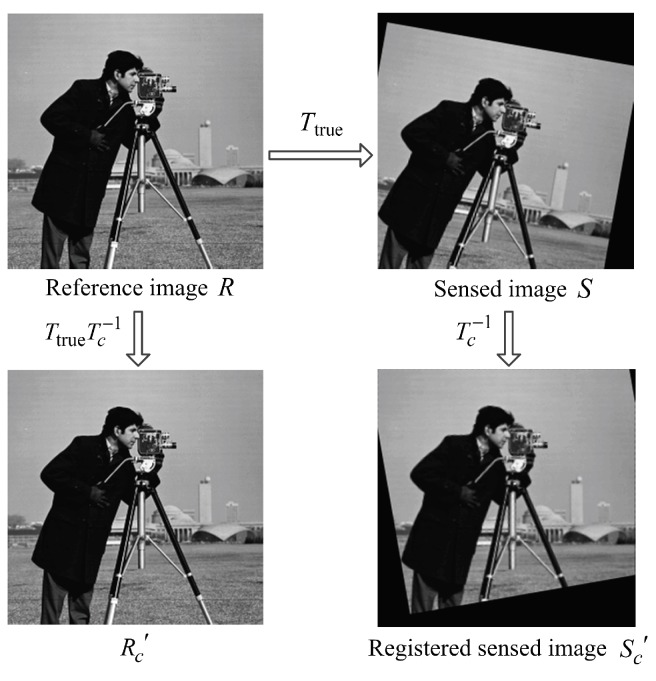
Relationship among *R*, *S*, ${R_{c}}^{\prime }$ and ${S_{c}}^{\prime }$.

Since the purpose of image registration is to align the reference image *R* and sensed image *S* to a single coordinate frame, one popular evaluation method for the registration result is to quantify the difference (usually quantified by Root-Mean-Squared Error (RMSE)) between *R* and the registered sensed image ${S_{c}}^{\prime }$ [31,32]. However, since ${S_{c}}^{\prime }$ is transformed from the sensed image *S*, which may have less information than *R* (*S* may be part of *R* or have lower resolution than *R* since *R* and *S* can be taken from different views or taken by different cameras), the difference between *R* and ${S_{c}}^{\prime }$ could be large even when the estimated transformation $T_{c}$ equals to the true transformation $T_{\mathrm{true}}$ from the reference image *R* to the sensed image *S*, as shown in Figure 7. Therefore, this kind of evaluation method is not accurate enough.

Another popular evaluation method is to quantify the difference between the reference image *R* and image ${R_{c}}^{\prime }$, which is transformed from *R* by the transformation matrix $T_{\mathrm{true}}T_{c}^{-1}$ [16,33], as shown in Figure 7. The mapping relationship between pixel at (v,w) in image *R* and pixel at $\left(v^{\prime },w^{\prime }\right)$ in image ${R_{c}}^{\prime }$ satisfies

(19) $$\left[v^{\prime }\;w^{\prime }\;1\right]=\left[v\;w\;1\right]\;T_{\mathrm{true}}T_{c}^{-1},$$

when the registration is absolutely accurate, $T_{c}=T_{\mathrm{true}}$ and ${R_{c}}^{\prime }=R$.

In this paper, we evaluate the registration performance by quantifying the difference between *R* and ${R_{c}}^{\prime }$ using AAID (average absolute intensity difference) [16]:

(20) $$\mathrm{AAID}\left(R,{R_{c}}^{\prime }\right)=\frac{1}{MN}\sum_{x=1}^{M}\sum_{y=1}^{N}\left|R\left(x,y\right)-{R_{c}}^{\prime }\left(x,y\right)\right|.$$

AAID$\left(R,{R_{c}}^{\prime }\right)$ is smaller when the registration result is better.

## 6. Experiments

To verify the performance of our new proposed image registration method, we provide experiments on noise-free images and noisy images, respectively. Image registration under the noisy condition is difficult since the noise pixels bring difficulties for keypoints’ detection and matching and reduce the accuracy for similarity measure. For the sparse method, experiment results based on BRISK [34], KAZE [15] and SURF [12] feature detection algorithms are provided for comparison. For the dense method, experiment results based on MI, PSNR and NCC similarity measures are provided for comparison. For the noisy image registration, the experiment result of RANRESAC (a recently proposed method for noisy image registration) [23] is also provided for comparison.

### 6.1. Sparse Image Registration Results

**Figure 8:**
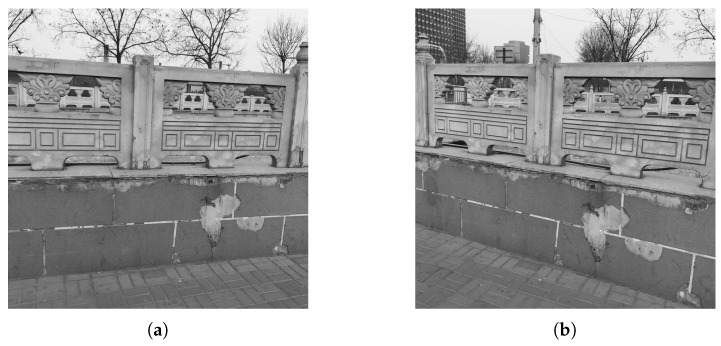
Fence image pair. (**a**) Reference image. (**b**) Sensed image.

We first do experiments on actual data to illustrate the effectiveness of the proposed method. The reference image and sensed image are taken from different cameras with different views, as shown in Figure 8. BRISK, KAZE and SURF feature detections are used for generating transformations $T_{1}$, $T_{2}$ and $T_{3}$, respectively. When deriving combined BBAs in Equation (17), an alternative fusion rule PCR6, which is more robust than Dempster’s rule [26], is also used for comparison.

**Figure 9:**
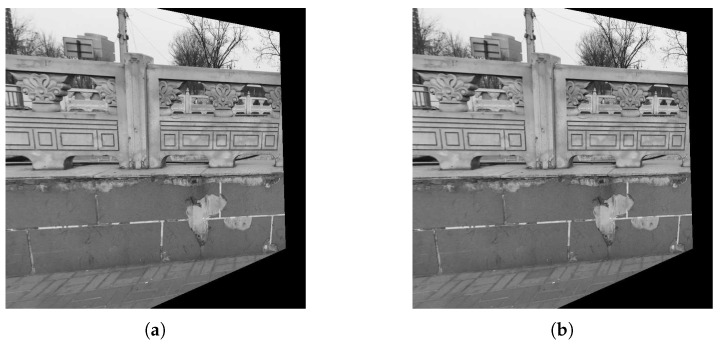
Registered results of the proposed methods for Fence image. (**a**) Dempster’s rule. (**b**) PCR6.

The registered results of the proposed method are illustrated in Figure 9. From Figure 9, the proposed method can successfully align the sensed image to the reference image illustrating that the proposed method is effective for actual data.

**Figure 10:**
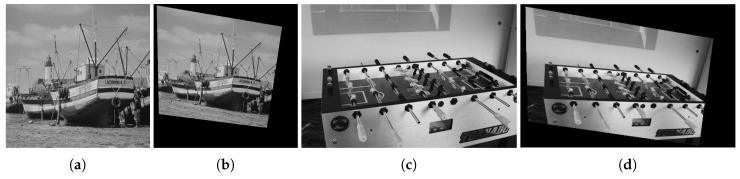
Boats image pair and Foosball image pair. (**a**) Boats reference image. (**b**) Boats sensed image. (**c**) Foosball reference image. (**d**) Foosball sensed image.

To quantify the accuracy of the registration results, the actual transformation between the reference image and sensed image is needed and we do experiments on simulated images. We first do experiments on Boats image (The reference image can be found at https://imagej.nih.gov/ij/images/boats.gif.) and Foosball image (sample image from the MATLAB), as shown in Figure 10.

**Figure 11:**
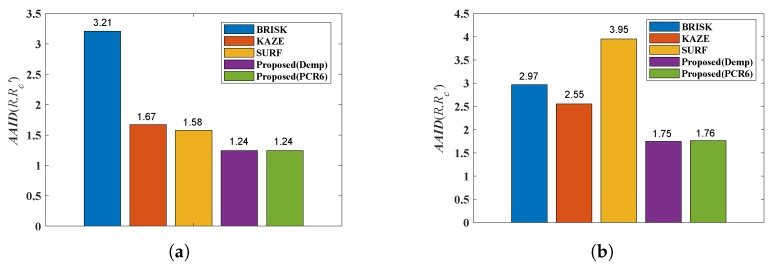
AAID evaluations of registration results for Boats image pair and Foosball image pair. (**a**) Boats. (**b**) Foosball.

The AAID evaluations of these registration results for Boats image and Foosball image are compared in Figure 11, where Demp represents the Dempster’s combination rule. According to Figure 11, the proposed fusion-based method achieves much better registration result (smaller AAID) than algorithms based on BRISK, KAZE or SURF feature detections, respectively.

**Figure 12:**
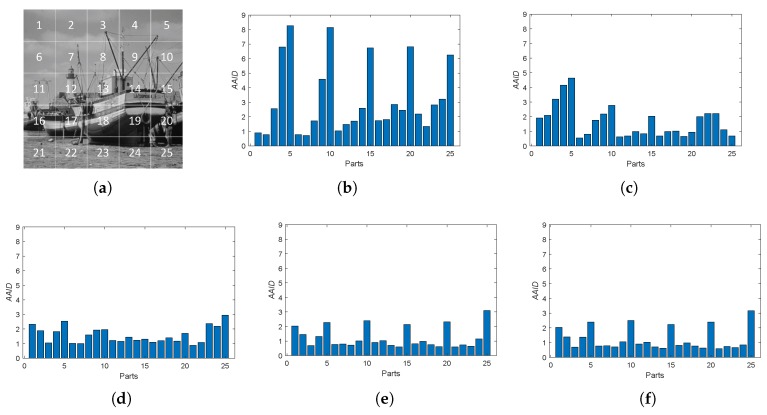
Spatial partition of the AAID evaluation for Boats image. (**a**) Partition method. (**b**) BRISK. (**c**) KAZE. (**d**) SURF. (**e**) Proposed (Demp). (**f**) Proposed (PCR6).

**Figure 13:**
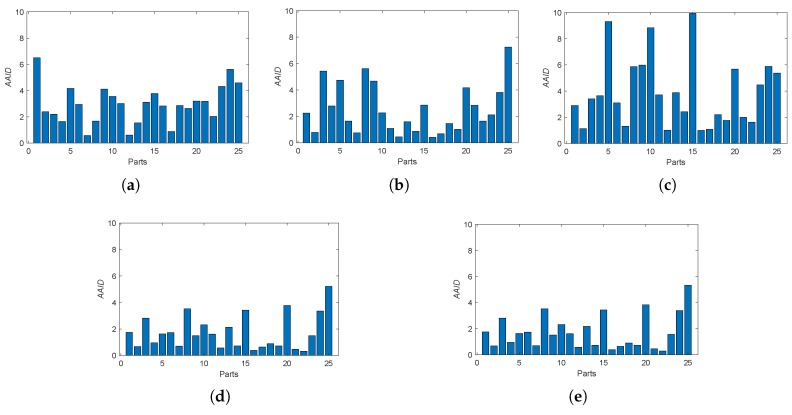
Spatial partition of the AAID evaluation for Foosball image. (**a**) BRISK. (**b**) KAZE. (**c**) SURF. (**d**) Proposed (Demp). (**e**) Proposed (PCR6).

Furthermore, we also analyzed the spatial partition of the AAID evaluation for each result by evenly dividing the reference image into 5×5 parts (as shown in Figure 12a) and calculating the AAID between the reference image and the registration result in each part. The AAID spatial partition results for Boats image and Foosball image are illustrated in Figure 12 and Figure 13, respectively. For Boats image, the AAID of BRISK and KAZE results varies significantly for different parts while the SURF result is relatively uniform; the proposed methods have low and similar AAID in most parts while the rightmost parts (parts 5, 10, 15, 20 and 25) have significant larger AAID. For Foosball image, the AAID spatial partition of all these results are uneven.

**Figure 14:**
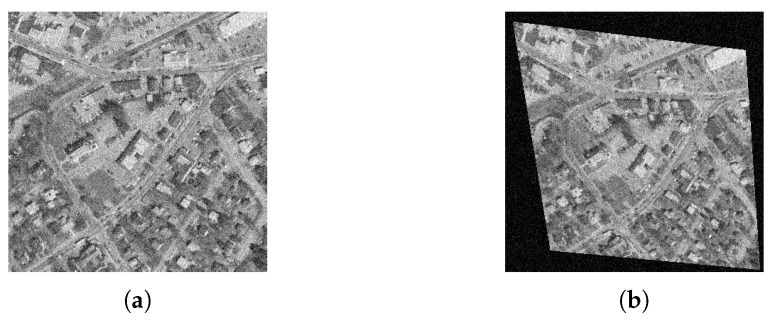
West Concord image pair. (**a**) Reference image. (**b**) Sensed image.

**Figure 15:**
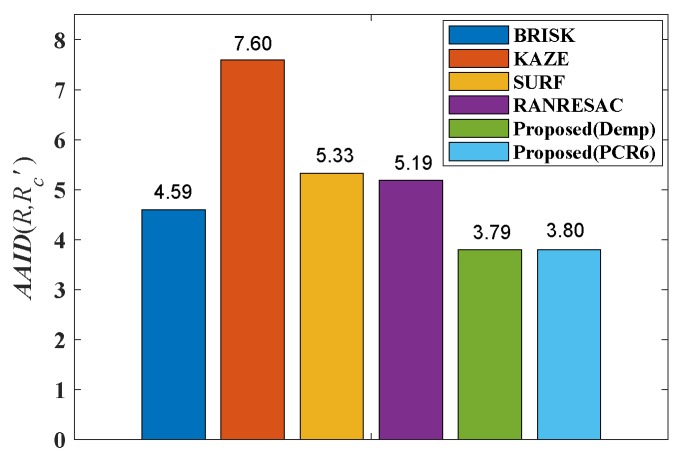
AAID evaluations of registration results for West Concord image pair.

**Figure 16:**
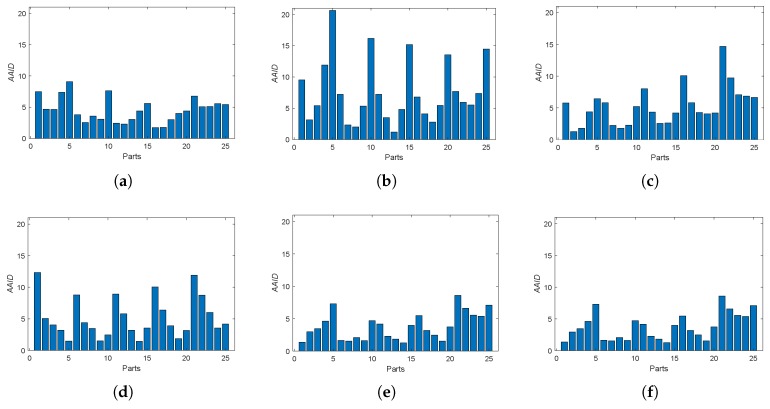
Spatial partition of the AAID evaluation for West Concord image. (**a**) BRISK. (**b**) KAZE. (**c**) SURF. (**d**) RANRESAC. (**e**) Proposed (Demp). (**f**) Proposed(PCR6).

Then, we consider the noisy image registration and do experiments on West Concord image pair (sample image from the MATLAB) with zero-mean Gaussian noise (variance is 0.01), as shown in Figure 14. The AAID evaluations for these registration results are compared in Figure 15, where the proposed fusion-based methods achieve better performance (smaller AAID) than RANTESAC and methods based on BRISK, KAZE and SURF feature detections, respectively. The spatial partition of the AAID evaluation for each result is illustrated in Figure 16, where the KAZE result is the most uneven one.

### 6.2. Dense Image Registration Results

**Figure 17:**
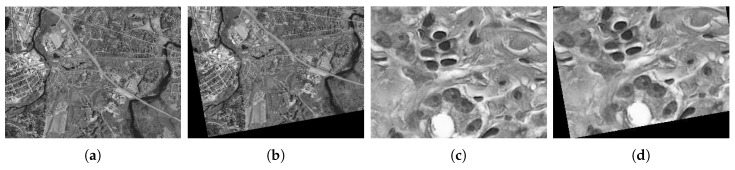
Concord image pair and Hestain image pair. (**a**) Concord reference image. (**b**) Concord sensed image. (**c**) Hestain reference image. (**d**) Hestain sensed image.

Since the optimization of dense registration is intractable when the solution space has high dimensions, we simplify the transformation model to rigid transformation here. The solution space for rigid model only has three dimensions: one for rotation and two for translations in horizontal and vertical directions, respectively. We first provide experiments on Concord image and Hestain image (sample images from the MATLAB) as shown in Figure 17, where the sensed image is transformed from the reference image through the rotation ($\theta =10^{\circ }$ in anticlockwise) and translation ($\left(t_{v},t_{h}\right)=\left(-10,5\right)$) successively.

**Figure 18:**
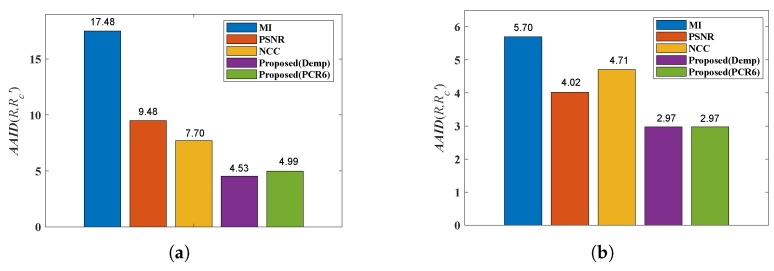
AAID evaluations of registration results for Concord image pair and Hestain image pair. (**a**) Concord. (**b**) Hestain.

**Figure 19:**
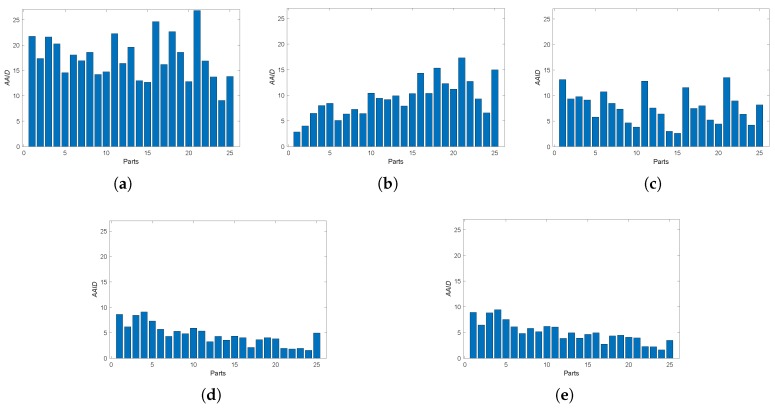
Spatial partition of the AAID evaluation for Concord image. (**a**) MI. (**b**) PSNR. (**c**) NCC. (**d**) Proposed (Demp). (**e**) Proposed(PCR6).

**Figure 20:**
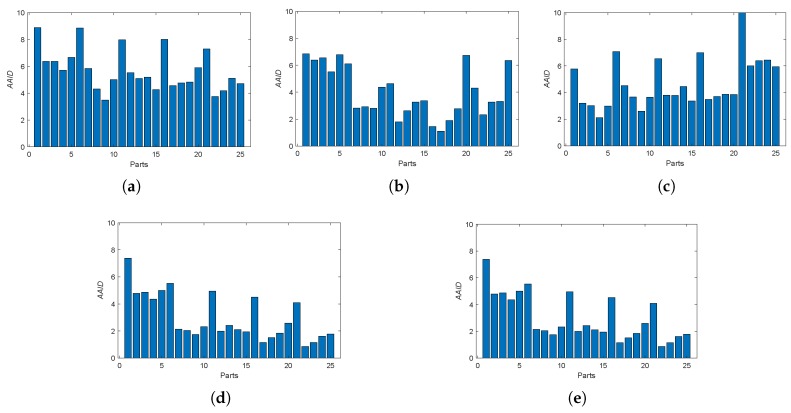
Spatial partition of the AAID evaluation for Hestain image. (**a**) MI. (**b**) PSNR. (**c**) NCC. (**d**) Proposed (Demp). (**e**) Proposed (PCR6).

In the proposed dense approach, MI, PSNR and NCC similarity measures are used for generating transformations $T_{1}$, $T_{2}$ and $T_{3}$, respectively. The AAID evaluations of these registration results for the Concord image and Hestain image are compared in Figure 18, where the proposed fusion-based methods achieve much better registration results (smaller AAID) than algorithms based on MI, PSNR or NCC similarities, respectively. The AAID spatial partition results for Concord image and Hestain image are illustrated in Figure 19 and Figure 20, respectively. For these two images, the AAID results of the proposed methods are smaller in the downside parts compared with those in upside parts.

**Figure 21:**
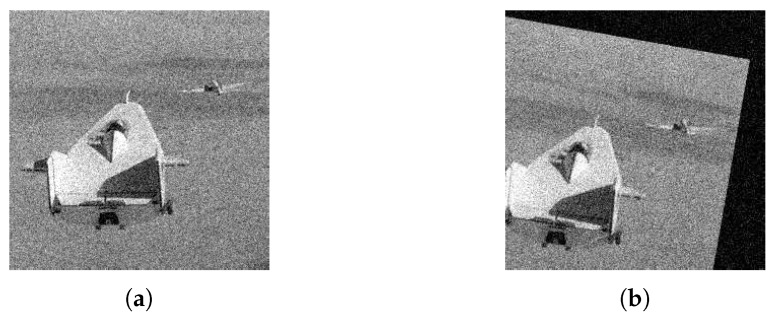
Lifting Body image pair. (**a**) Reference image. (**b**) Sensed image.

Then, we consider the noisy image condition and implement experiments on Lifting Body image pair (sample image from the MATLAB) with zero-mean Gaussian noise (variance is 0.01), as shown in Figure 21. The sensed image is transformed from the reference image through the rotation ($\theta =-10^{\circ }$) and translation ($\left(t_{v},t_{h}\right)=\left(-10,5\right)$), successively.

**Figure 22:**
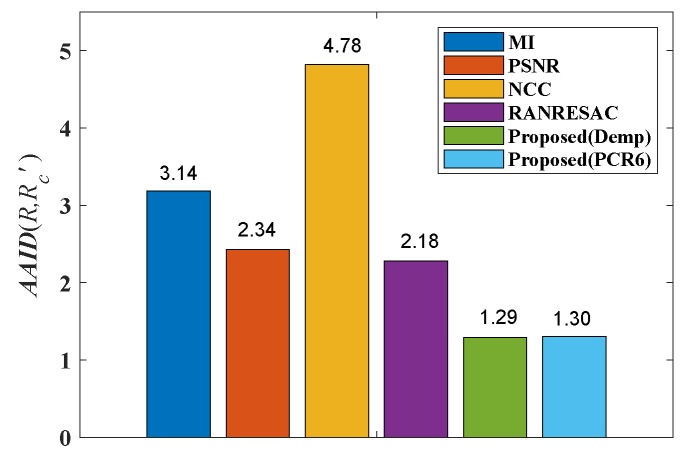
AAID evaluations of registration results for Lifting Body image pair.

**Figure 23:**
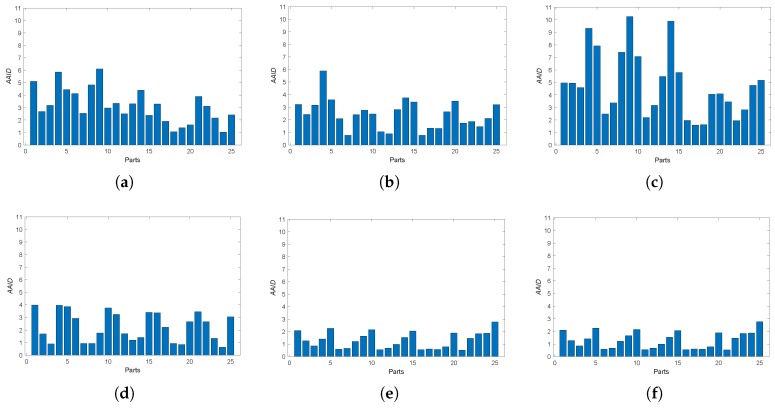
Spatial partition of the AAID evaluation for Lifting Body image. (**a**) MI. (**b**) PSNR. (**c**) NCC. (**d**) RANRESAC. (**e**) Proposed (Demp). (**f**) Proposed (PCR6).

The AAID evaluations for these registration results are compared in Figure 22 and the spatial partition of the AAID evaluation for each result is illustrated in Figure 23, From these two figures, the proposed fusion-based methods achieve better performance and the rightmost parts (parts 5, 10, 15, 20 and 25) have larger AAID than other parts.

According to all the experiments, the proposed fusion-based methods achieve better registration results than those prevailing ones (BRISK, KAZE, SURF, MI, PSNR and NCC). For noisy image registration, the proposed methods also obtain better performance than RANRESAC. This indicates that the theory of belief function can well deal with the uncertainty brought by the selection of keypoint detection algorithms or similarity measures, and the jointly use of the different keypoint detections or similarity measures is effective. Furthermore, from the above provided experiments one sees that the choice of combination rule does not affect the registration performance that much.

### 6.3. Computational Cost

The computational cost is an important criterion to evaluate an algorithm. We counted the computational costs of the above sparse algorithms and dense algorithms for Cameraman image (Figure 5a) on a Windows 10 Enterprise system equipped with Intel Core i7-7700HQ CPU at 2.80 GHz and 16.00 GB RAM. The platform is MATLAB R2018a. The average execution time comparisons for the sparse algorithms and dense algorithms are provided in Table 2 and Table 3, respectively. Each average execution time is calculated from 100 runs of experiments.

From Table 2 and Table 3, the dense algorithms need more execution time than the sparse algorithms. Furthermore, since the proposed fusion-based method combines the registration transformations generated from the three sparse methods (BRISK, KAZE and SURF) or the three dense methods (PSNR, MI and NCC) and these three methods can be parallely executed, the execution time of the proposed fusion-based method is longer than the most time-consuming one among the three methods.

### 6.4. Discussion of BBA Generation

The BBA generated in Equation (16) is Bayesian BBA, where all its focal elements are singletons. People in the community of belief function theory may prefer to use the compound focal elements, which usually seems better than only using singletons in Bayesian BBAs. We have also designed experiments of generating non-Bayesian BBAs for image registration using FCOWA-ER (Fuzzy-Cautious Ordered Weighted Averaging with Evidential Reasoning) [35] method. In detail, when multiple image information (image’s intensities, edges and phase angle) are simultaneously considered, image registration can be viewed as a multi-criteria decision making problem. FCOWA-ER (Fuzzy-Cautious Ordered Weighted Averaging with Evidential Reasoning) [35] is a decision making approach under multi-criteria with uncertainty and it generates non-Bayesian BBAs using α-cut method (The α-cut method used in FCOWA-ER boils down to the Dubois and Prade allocation [36] in this case) when modeling uncertainties. According to the experimental results, non-Bayesian BBAs obtain similar registration results with Bayesian BBAs. Since Bayesian BBAs are easier to generate than non-Bayesian BBAs, we recommend Bayesian BBAs for image registration and do not provide the non-Bayesian BBA based method in this work.

## 7. Conclusions

In this paper, we proposed a new image registration algorithm based on evidential reasoning. The uncertainty encountered in image registration is taken into account and modeled by belief functions. Image information at different levels are jointly used to achieve a more effective registration. Experimental results show that the proposed algorithm can improve the precision of image registration.

The generation of BBA is crucial in evidential reasoning and most methods are proposed based on applications. In this paper, we generate BBAs from three different image information, i.e., intensity, edge and phase angle. In future work, other image information, such as texture feature and gradient feature, will also be considered and jointly used in image registration. Furthermore, we will attempt to apply the proposed method to color image registration. Different color channels of the color image provide different image information and can be jointly used in image registration. We will also focus on the comparison with the state-of-the-art approaches based on convolutional neural networks (CNN).

## Figures and Tables

**Table 1:** Projective transformation and its two specializations.

Similarity	Affine	Projective
$\left[\begin{array}{ccc} s\cos\theta & s\sin\theta & 0\\ -s\sin\theta & s\cos\theta & 0\\ t_{31} & t_{32} & 1 \end{array}\right]$	$\left[\begin{array}{ccc} t_{11} & t_{12} & 0\\ t_{21} & t_{22} & 0\\ t_{31} & t_{32} & 1 \end{array}\right]$	$\left[\begin{array}{ccc} t_{11} & t_{12} & t_{13}\\ t_{21} & t_{22} & t_{23}\\ t_{31} & t_{32} & t_{33} \end{array}\right]$
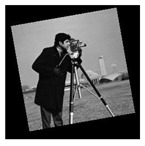	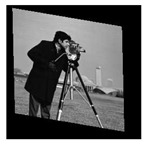	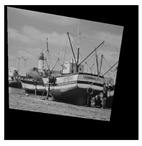

**Table 2:** Average execution time comparison for sparse algorithms (unit second).

Method	Noise-Free Images	Noisy Images
BRISK	0.2847	0.2832
KAZE	0.1348	0.1304
SURF	0.0431	0.0437
RANRESAC	–	6.2648
Proposed (Demp)	0.3934	0.3933
Proposed (PCR6)	0.3938	0.3989

**Table 3:** Average execution time comparison for dense algorithms (unit second).

Method	Noise-Free Images	Noisy Images
MI	16.7622	16.4789
PSNR	12.8583	13.4214
NCC	14.7187	15.1050
Proposed (Demp)	17.0734	16.7945.
Proposed (PCR6)	17.0812	16.8729

## References

[1] Meher B., Agrawal S., Panda R., Abraham A. (2019). A survey on region based image fusion methods. Inf. Fusion.

[2] Krylov V.A., Moser G., Serpico S.B., Zerubia J. (2016). False discovery rate approach to unsupervised image change detection. IEEE Trans. Image Process..

[3] Torabi A., Massé G., Bilodeau G. (2012). An iterative integrated framework for thermal-visible image registration, sensor fusion, and people tracking for video surveillance applications. Comput. Vis. Image Underst..

[4] Zhang G., Wu Q., Wang T., Zhao R., Deng M., Jiang B., Li X., Wang H., Zhu Y., Li F. (2018). Block Adjustment without GCPs for Chinese Spaceborne SAR GF-3 Imagery. Sensors.

[5] Saygili G., Staring M., Hendriks E.A. (2016). Confidence estimation for medical image registration based on stereo confidences. IEEE Trans. Med. Imaging.

[6] Zitová B., Flusser J. (2003). Image registration methods: A survey. Image Vis. Comput..

[7] Guo X., Xu Z., Lu Y., Pang Y. An Application of Fourier-Mellin Transform in Image Registration. Proceedings of the International Conference on Computer and Information Technology.

[8] Ask E., Enqvist O., Svärm L., Kahl F., Lippolis G. Tractable and reliable registration of 2D point sets. Proceedings of the 13th European Conference on Computer Vision.

[9] Harris C., Stephens M. A combined corner and edge detector. Proceedings of the 4th Alvey Vision Conference.

[10] Rosten E., Drummond T. Machine learning for high-speed corner detection. Proceedings of the 9th European Conference on Computer Vision.

[11] Lowe D.G. Object recognition from local scale-invariant features. Proceedings of the International Conference on Computer Vision.

[12] Bay H., Tuytelaars T., Gool L.V. (2008). Surf: Speeded up robust features. Comput. Vis. Image Underst..

[13] Tola E., Lepetit V., Fua P. (2010). Daisy: An efficient dense descriptor applied to wide baseline stereo. IEEE Trans. Pattern Anal. Mach. Intell..

[14] Rublee E., Rabaud V., Konolige K., Bradski G. ORB: An efficient alternative to SIFT or SURF. Proceedings of the IEEE International Conference on Computer Vision.

[15] Alcantarilla P.F., Bartoli A., Davison A.J. KAZE Features. Proceedings of the European Conference on Computer Vision.

[16] Goshtasby A.A. (2012). Image Registration: Principles, Tools and Methods.

[17] Santini S., Jain R. (1999). Similarity measures. IEEE Trans. Pattern Anal. Mach. Intell..

[18] Spearman C. (2010). The proof and measurement of association between two things. Int. J. Epidemiol..

[19] Shafer G. (1976). A Mathematical Theory of Evidence.

[20] Han D., Dezert J., Li S., Han C., Yang Y. Image registration based on evidential reasoning. Proceedings of the 16th International Conference on Information Fusion.

[21] Fischler M.A., Bolles R.C. (1987). Readings in Computer Vision: Issues, Problem, Principles, and Paradigms.

[22] Torr P.H.S., Murray D.W. (1997). The development and comparison of robust methods for estimating the fundamental matrix. Int. J. Comput. Vis..

[23] Nakazawa A. Noise stable image registration using random resample consensus. Proceedings of the 23rd International Conference on Pattern Recognition.

[24] Jain P., Kar P. (2017). Nonconvex Optimization for Machine Learning. Found. Trends Mach. Learn..

[25] Pham D.T. (2000). Intelligent Optimisation Techniques.

[26] Smarandache F., Dezert J. (2015). Advances and Applications of DSmT for Information Fusion: Collected Works IV.

[27] Smets P. (1994). The transferable belief model. Artif. Intell..

[28] Han D., Dezert J., Duan Z. (2016). Evaluation of probability transformations of belief functions for decision making. IEEE Trans. Syst. Man Cybern. Syst..

[29] Gonzalez R.C., Woods R.E. (2008). Digital Image Processing.

[30] Canny J. (1986). A computational approach to edge detection. IEEE Trans. Pattern Anal. Mach. Intell..

[31] Ferreira D.P.L., Ribeiro E., Barcelos C.A.Z. (2018). A variational approach to non-rigid image registration with bregman divergences and multiple features. Pattern Recognit..

[32] Xia X., Dang G., Yao Y., Liang J. (2017). Image registration model and algorithm for multi-focus images. Pattern Recognit. Lett..

[33] Pluim J.P.W., Likar B., Gerritsen F.A. (2006). Biomedical Image Registration.

[34] Leutenegger S., Chli M., Siegwart R.Y. BRISK: Binary Robust invariant scalable keypoints. Proceedings of the IEEE International Conference on Computer Vision.

[35] Han D., Dezert J., Tacnet J.M., Han C. A fuzzy-cautious OWA approach with evidential reasoning. Proceedings of the International Conference on Information Fusion.

[36] Dubois D., Prade H. (1988). Representation and Combination of Uncertainty with Belief Functions and Possibility Measures. Comput. Intell..

